# Understanding immune phenotypes in human gastric disease tissues by multiplexed immunohistochemistry

**DOI:** 10.1186/s12967-017-1311-8

**Published:** 2017-10-12

**Authors:** Le Ying, Feng Yan, Qiaohong Meng, Xiangliang Yuan, Liang Yu, Bryan R. G. Williams, David W. Chan, Liyun Shi, Yugang Tu, Peihua Ni, Xuefeng Wang, Dakang Xu, Yiqun Hu

**Affiliations:** 1 Faculty of Medical Laboratory Science, Ruijin Hospital, School of Medicine, Shanghai Jiao Tong University, 227 Chongqing Road South, Shanghai, 200025 China; 2 Institute of Ageing Research, Hangzhou Normal University School of Medicine, Hangzhou, China; 3 Department of Tea Science, Zhejiang University, Hangzhou, China; 4 Department of General Surgery, Shanghai Jiao Tong University Affiliated First People’s Hospital, Shanghai, China; 5 Hudson Institute of Medical Research, Department of Molecular and Translational Science, Monash University, Clayton, VIC Australia; 6 Department of Obstetrics and Gynaecology, LKS Faculty of Medicine, The University of Hong Kong, Hong Kong SAR, P. R. China; 7 Department of Microbiology and Immunology, Nanjing University of Chinese Medicine, Nanjing, 210046 China; 8 Cell Signaling Technology, Inc., Asia Pacific, Danvers, USA; 9 Department of Laboratory Medicine, Ruijin Hospital, Shanghai Jiaotong University School of Medicine, Shanghai, China

**Keywords:** Human gastric disease, Immune phenotypes, Multiplexed immunohistochemistry

## Abstract

**Background:**

Understanding immune phenotypes and human gastric disease in situ requires an approach that leverages multiplexed immunohistochemistry (mIHC) with multispectral imaging to facilitate precise image analyses.

**Methods:**

We developed a novel 4-color mIHC assay based on tyramide signal amplification that allowed us to reliably interrogate immunologic checkpoints, including programmed death-ligand 1 (PD-L1), cytotoxic T cells (CD8^+^T) and regulatory T cells (Foxp3), in formalin-fixed, paraffin-embedded tissues of various human gastric diseases. By observing cell phenotypes within the disease tissue microenvironment, we were able to determine specific co-localized staining combinations and various measures of cell density.

**Results:**

We found that PD-L1 was expressed in gastric ulcer and in tumor cells (TCs), as well as in tumor-infiltrating immune cells (TIICs), but not in normal gastric mucosa or other gastric intraepithelial neoplastic tissues. Furthermore, we found no significant reduction in CD8^+^T cells, whereas the ratio of CD8^+^T:Foxp3 cells and CD8^+^T:PD-L1 cells was suppressed in tumor tissues and elevated in adjacent normal tissues. An unsupervised hierarchical analysis also identified correlations between CD8^+^T and Foxp3^+^ tumor-infiltrating lymphocyte (TIL) densities and average PD-L1 levels. Three main groups were identified based on the results of CD8^+^T:PD-L1 ratios in gastric tumor tissues. Furthermore, integrating CD8^+^T:Foxp3 ratios, which increased the complexity for immune phenotype status, revealed 6–7 clusters that enabled the separation of gastric cancer patients at the same clinical stage into different risk-group subsets.

**Conclusions:**

Characterizing immune phenotypes in human gastric disease tissues via multiplexed immunohistochemistry may help guide PD-L1 clinical therapy. Observing unique disease tissue microenvironments can improve our understanding of immune phenotypes and cell interactions within these microenvironments, providing the ability to predict safe responses to immunotherapies.

**Electronic supplementary material:**

The online version of this article (doi:10.1186/s12967-017-1311-8) contains supplementary material, which is available to authorized users.

## Background

Host and tumor tissues undergo extensive immune interactions, and the ability of the tumor to evade immune recognition often determines clinical outcomes. Immunotherapy has recently emerged as a novel approach in treating solid tumors. Tumor-infiltrating lymphocytes (TIL) play an essential role in mediating the response to immunotherapy and affect clinical outcomes in many cancer subtypes. The presence of TILs within the tumor microenvironment has been linked to better prognosis in gastric cancer [1]. “Immunologic checkpoints” expressed on cells in the tumor microenvironment or on the surface of tumor cells (TCs) can promote immune escape by inducing apoptosis in immune effector cells. One such immune checkpoint is programmed cell death protein 1 (PD-1). The binding of PD-1 to its ligand programmed cell death 1 ligand 1 (PD-L1) leads to a blockade of kinases involved in T cell activation. PD-L1 expression on TCs is associated with worse prognosis in many malignancies [2]. Many recent studies have reported that the response to immunotherapy primarily depends on the expression of PD-L1 in the tumor microenvironment. These findings suggest that the response to anti-PD-1 hinges on pre-existing anti-tumor immune response and that anti-PD-1 acts to free the CD8 T cells from inhibition to exert their anti-tumor activities [3]. Furthermore, naturally occurring TILs can be detected in various solid tumors, even in metastatic stages [4]. Immunosuppressive mechanisms within the tumor microenvironment have been associated with the failure of TILs [5]. Biomarkers of immune phenotypes are most commonly identified using immunohistochemistry (IHC). Studies using quantitative IHC have identified CD8^+^ T cell infiltration as an important prognostic factor in predicting outcomes in patients with gastric cancer [6]. CD8^+^ T cells are regulated by regulatory T cells (Tregs). Among all Tregs, Foxp3-expressing Tregs are well known to play a critical role in tumor immune evasion [7], which has been reported in a wide array of human malignancies including our study in gastric cancer [7, 8]. Furthermore, upregulation of Tregs is associated with significantly reduced CD8^+^ T cell infiltration of tumors and with worse outcomes for cancer patients [9]. However, the clinical implications of immunosuppressive processes related to immunologic checkpoints (PD-L1, CD8, and Foxp3) in tumors or immune cells in the tumor microenvironment remain controversial, and the potential use of these checkpoints as prognostic markers requires further study.

Tissue microarrays (TMAs) are commonly employed in clinical and basic-science research, it is a powerful tool for undertaking large-scale tissue-based biomarker studies [10] and has been widely used in many studies that involved in immune infiltrates studies [11, 12]. Tissue sections from TMAs also offer the opportunity to understand a patient’s disease condition, to make better prognostic evaluations and to select optimum treatments. But tissues are often assessed primarily based on visual analysis of one or two molecules, image analysis is starting to address the variability of human samples. This is in contrast to measure characteristics such as parameters revealed through co-expression, spatial relationships, heterogeneity, and low abundance molecules. Those factors are understood to be critical to develop effective therapeutic strategies [13]. So far, TMA performance across multiple biomarkers has not been systematically explored. Here the multiplex immunohistochemistry (mIHC) was employed in our study for mapping the tumor microenvironment. This technology allows us to explore the relationship between various cell types in the peritumoral and intratumoral compartments through more comprehensive and efficient analysis of the tumor microenvironment. mIHC quantifies the change in expression or state of specific biomarkers and examines their impact on patient disease status for both mechanistic and diagnostic studies, potentially advancing our understanding of immune phenotypes and cell interactions in the microenvironment while also providing better direct patient treatment based on individual tumor microenvironment responses to immunotherapy, offering improved response rates with gastric disease [14]. Here we developed a 4-color multispectral quantitative fluorescent immunohistochemistry methods to detect CD8, Foxp3 and PD-L1 simultaneously.

## Methods

### Sample and tissue microarray preparation

Gastric cancer tissues were obtained from Shanghai Jiao Tong University, Ruijin Hospital. These tissues were formalin-fixed and paraffin-embedded for use. All of the protocols using human specimens were approved by Shanghai Jiao Tong University, and informed consent was obtained from all of the subjects. One TMA (TMA 1) included 30 gastric cancer tissues from patients, but 7 samples were excluded due to incompleteness. Another TMA (TMA 2) contained 30 various gastric disease samples (6 normal tissues, 9 gastric ulcer tissues, 1 gastric polyp tissue, 3 gastric intraepithelial neoplasia tissues, 6 gastric carcinoma tissues and 6 normal adjacent tissues), of which 2 normal tissues, 1 gastric ulcer tissue, 1 gastric polyp tissue and 1 gastric carcinoma tissue were also excluded due to incompleteness. The remaining 49 tissues were designated as Sample 1 to Sample 49 for further analysis. Detailed information is shown in Additional file 1: Table S1. Two TMAs were made based on pathology diagnosis of each tissues. The blocks were assembled and a surgical pathologist reviews the H&E slide for each case. The pathologist then circled the area of the block, localizing a representative tumor region from which a core will be extracted for our TMAs. Then we applied H&E staining on the two TMAs to validate pathology type of each tissue on each TMA, and the results of H&E staining was shown in Additional file 1: Figure S1. The core diameter on each TMA in this study is 2 mm, which is much larger than the normal TMA core (0.6 mm), to provide more representative tissues on TMA.

### Immunohistochemistry

Gastric cancer paraffin blocks were processed into four-micrometer-thick sections and mounted on slides for staining. Each slide contained 2 samples of the same tissue. First, the slides were deparaffinized in xylene using a graded ethanol series (75, 50, 25%). Antigen recovery was performed by microwaving the samples for 15 min in citrate buffer (pH 6.0), and the slides were cooled for 30 min. Endogenous peroxidases were then blocked by treating the tissues with 3% H_2_O_2_ for 10 min. Then, the tissues were incubated with blocking serum for 20 min at room temperature. Afterwards, the slides were incubated overnight with CD8^+^T (1:500 dilution in SignalStain Antibody Diluent), PD-L1 (1:250 dilution in SignalStain Antibody Diluent), and Foxp3 (1:350 dilution in SignalStain Antibody Diluent) primary antibodies (Cell Signaling, USA) at 4 °C and incubated with horseradish peroxidase (HRP) (Vectastain ABC kit, USA) for 30 min the next day. The slides were incubated with ABC reagent (Vectastain ABC kit, USA) for another 30 min at room temperature, washed with TBST, and stained with 3, 3′-diaminobenzidine (DAB). Finally, the slides were counterstained with hematoxylin and mounted with coverslips in DPX (Sigma, USA) for imaging. All staining preparations included a no primary antibody control, details are described by a previous study [15].

### Multiplex immunohistochemistry

For mIHC staining, a PD-L1, Foxp3, and CD8a multiplex IHC antibody panel kit (Cell Signaling, USA) and Opal 4-color fluorescent IHC kit (PerkinElmer, USA) were used [13]. First, the concentration and the order of the three antibodies were optimized, and the spectral library was built based on the single-stained slides. The slides were first deparaffined by xylene and ethanol (different concentrations) and antigen retrieval was performed by microwave. After incubating with 3% H_2_O_2_ (freshly made) for 10 min, the tissues were blocked in blocking buffer for another 10 min at room temperature. Then the tissues were incubated by primary antibody (Cell signaling, USA), secondary-HRP (Cell signaling, USA) and Opal working solution (PerkinElmer, USA). The slides were mounted with ProLong Gold Antifade Reagent with DAPI (Cell signaling, USA). All the slides were scanned using a Nikon C1 confocal microscope (Nikon, Japan), and images were analyzed by ImageJ software (National Institutes of Health, USA). The positive cell numbers of CD8 and Foxp3 were calculated by ImageJ software and the average intensity of PD-L1 was also analyzed by ImageJ software. The Analyze Particles tool in ImageJ was used to count the total number of positive CD8 and Foxp3 cells. Other details are described by a previous study [16]. The results were confirmed by two experienced pathologists blinded to the clinicopathological parameters (Additional file 1: Figure S2). The detailed protocol is described in the Additional file 1: Methods.

### Statistical analysis

The statistical analysis was carried out using the Kruskal–Wallis test for nonparametric analysis in GraphPad Prism (Version 5.0), and differences were considered statistically significant at p < 0.05. Heatmaps and hierarchical clusters were generated in Rstudio (Version 3.2.0). Pearson correlation was performed by SPSS version 16.0 for Windows.

## Results

### Comparison of immunohistochemistry and mIHC to understand immune phenotypes in human gastric cancer

**Fig. 1:**
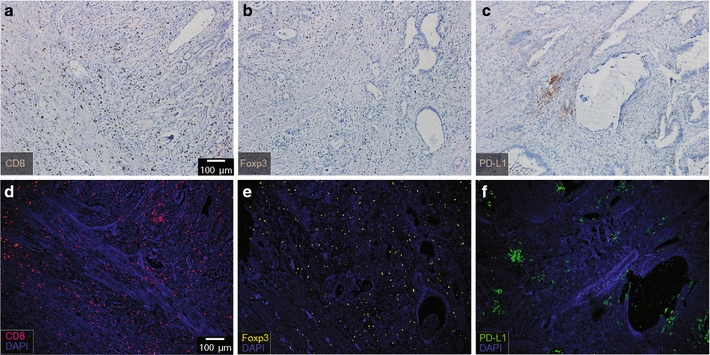
Comparison between IHC staining and 4-color mIHC staining of paraffin-embedded gastric cancer tissue (× 10). **a**–**c** IHC staining of CD8 (1:500), Foxp3 (1:350) and PD-L1 (1:250). **d**, **e** 4-color mIHC staining of CD8a (1:500), Foxp3 (1:350) and PD-L1 (1:250). DAPI was used to visualize nuclei (blue color), FITC corresponds to PD-L1 (green color), Cy3 represents Foxp3 (yellow color), and Cy5 indicates CD8 (red color)

**Fig. 2:**
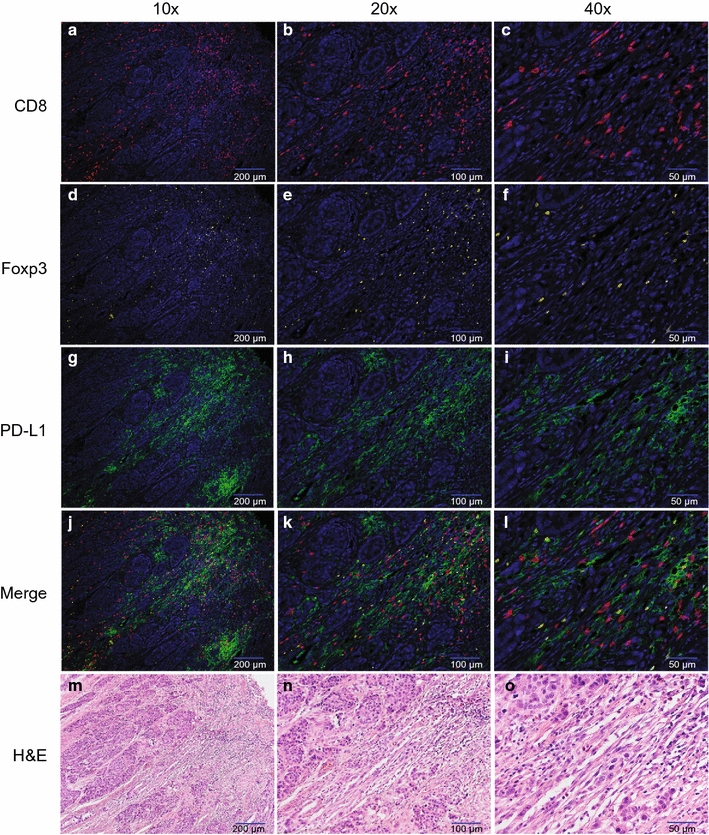
mIHC images with individual channels in gastric cancer tissues. **a**–**c** CD8 expression (red color) in gastric cancer tissues (**a** 10 × image; **b** × 20 image; **c** × 40 image). **d**–**f** Foxp3 expression (yellow color) in gastric cancer tissues (**d** × 10 image; **e** × 20 image; **f** × 40 image). **g**–**i** PD-L1 expression (green color) in gastric cancer tissues (**g** × 10 image; **h** × 20 image; **i** × 40 image). **j**–**l** Merge pictures of mIHC images (**j** × 10 image; **k** × 20 image; **l** × 40 image). **m**–**o** H&E staining images (**m** × 10 image; **n** × 20 image; **o** × 40 image). DAPI was used to visualize nuclei (blue color), FITC was used to visualize PD-L1 (green color), Cy3 indicates Foxp3 (yellow color), and Cy5 corresponds to CD8 (red color)

We hypothesized that analyzing cell–cell relationships via mIHC will provide a more complete view of tumor microenvironments than traditional IHC. To establish mIHC as a method for evaluating immune phenotypes in the microenvironments of human gastric disease, we first compared traditional IHC with more advanced 4-color mIHC. First, formalin-fixed, paraffin-embedded tissues from gastric cancer patients were stained by IHC. After optimization of the antibody dilution and antigen recovery conditions, CD8^+^T, Foxp3 and PD-L1 were detected in gastric cancer tissues. CD8^+^T and Foxp3 were expressed in TILs, while PD-L1 was expressed in both tumors and TILs (Fig. 1a, b). PD-L1 showed membrane-enriched expression (Fig. 1c). Then, mIHC was applied on the same paraffin tissues, and the mIHC experimental conditions were optimized individually with certain detection parameters and compared with IHC. CD8^+^T, Foxp3 and PD-L1 expression patterns were very similar with the IHC results in the same tissue sections (Fig. 1d–f). Clearer images of tumor microenvironments were observed using 4-color multispectral immunofluorescent staining. As shown in Fig. 2, CD8 was observed to express on the surface of lymphocytes (Fig. 2a–c) while Foxp3 expressed in the nuclear of the lymphocytes (Fig. 2b–f). PD-L1 was observed in the membrane and/or cytoplasm of tumor and stromal cells (Fig. 2g–i). PD-L1 is a member of the B7 family of cell surface ligands that regulate T cell activation and immune responses [17]. The PD-L1 ligand binds the PD-1 transmembrane receptor and inhibits T cell activation. Thus, analyzing multiple cells markers (CD8^+^T, Foxp3 and PD-L1) (Fig. 2j–l) allows us to assess spatial and interaction relationships, which provide valuable information for their relative function in this disease. To better understand the pathology type of the tissue, we applied H&E staining on the paraffin tissues to validate the pathology type of gastric cancer samples (Fig. 2m–o). Based on the results of H&E staining (Fig. 2m–o) and 4-color mIHC staining (Fig. 2j–l), the gastric cancer tissue in Fig. 2 is the typical representative sample that showed CD8, Foxp3 and PD-L1 expressed in tumor-infiltrated lymphoid tissues.

### Differential expressions pattern of CD8^+^T, Foxp3 and PD-L1 in gastric diseases

**Fig. 3:**
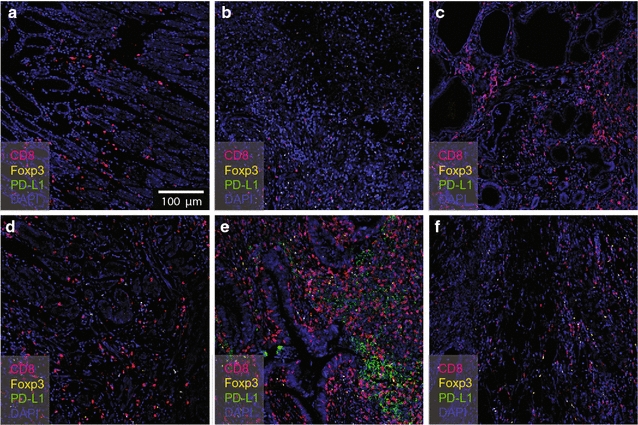
4-color mIHC images of different samples (× 20). **a** Normal gastric mucosa tissue (Sample 24). **b** Gastric ulcer tissue (Sample 30). **c** Gastric intraepithelial neoplasia tissue (Sample 38). **d** Normal adjacent tissue (Sample 44). **e** Gastric cancer tissue (Sample 43). **f** Gastric cancer tissue (Sample 47)

To assess differences in the expression of CD8^+^T, Foxp3^+^ and PD-L1, two TMA slides were used to compare the differences between gastric cancer tissues and other common gastric diseases, such as gastric ulcers. The two TMA slides were stained under the same experimental conditions after optimization as described above. There were totally 49 samples of different gastric diseases with 28 gastric cancer tissues, 4 normal gastric tissues, 8 gastric ulcer tissues, 3 gastric intraepithelial neoplasia tissues and 6 tumor normal adjacent tissues in two TMA slides (Additional file 1: Table S1). Six representative images are shown in Fig. 3. In the normal gastric mucosa tissue (Fig. 3a), gastric ulcer tissue (Fig. 3b), gastric intraepithelial neoplasia tissue (Fig. 3c) and normal adjacent tissue (Fig. 3d), CD8^+^T cells displayed different patterns depending on the disease. However, different patterns of immune infiltration were observed in gastric tissues from diseases, including normal gastric mucosa, gastric ulcer, gastric intraepithelial neoplasia and gastric cancer. Even in gastric cancer, two distinct patterns of immune infiltrate were observed in our cohort: patients with extensive immune infiltrates, which here presented extensive PD-L1 and Foxp3^+^ expression (Fig. 3e), and those with limited immune infiltrate, whose tissue presented little PD-L1 and limited Foxp3 expression (Fig. 3f). This result was consistent with that of Wu et al., who reported that approximately 42.2% of gastric carcinoma tissues can be detected by PD-L1 [18].

### CD8^+^T, Foxp3 and PD-L1 expression correlated with immune phenotypes in different gastric diseases

**Fig. 4:**
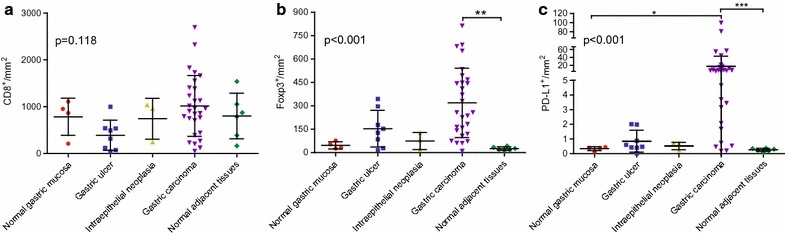
Quantification of the CD8^+^T, Foxp3^+^, and PD-L1^+^ cells in 49 samples. **a** Number of CD8^+^ cells per mm^2^. **b** Number of Foxp3^+^ cells per mm^2^. **c** Average intensity of PD-L1 per mm^2^. Data were analyzed using the Kruskal–Wallis test (nonparametric analysis). *p < 0.05, **p < 0.01, ***p < 0.001

To quantitatively compare the different levels of CD8^+^T, Foxp3 and PD-L1 expression between various types of gastric disease and cancers, all the samples on the TMA slides were scanned using a Nikon confocal microscope. The numbers of CD8^+^ T cells and Foxp3^+^ cells per mm^2^ were analyzed and calculated in ImageJ, along with the average PD-L1 intensity. All images were also scored by two experienced pathologists, yielding similar results (Additional file 1: Figure S2). As summarized in Fig. 4, no differences were observed between different tissues regarding the number of CD8^+^ T cells. However, the number of Foxp3 cells was significantly (p < 0.01) higher in gastric cancer tissues (319 Foxp3^+^ cells per mm^2^) than in normal adjacent tissues (25 Foxp3^+^ cells per mm^2^). PD-L1 expression in gastric cancer tissues (17.41 PD-L1 average intensity per mm^2^) was also significantly higher than in normal gastric mucosa tissues (0.34 PD-L1 average intensity per mm^2^) and normal adjacent tissues (0 PD-L1 average intensity per mm^2^). Interestingly, PD-L1 expression was also increased in gastric ulcer tissues (Fig. 4 **)**, possibly due to *Helicobacter pylori* infection [19].

### The ratios of CD8^+^T:Foxp3^+^ and CD8^+^T:PD-L1 cells were suppressed in tumor tissues

**Fig. 5:**
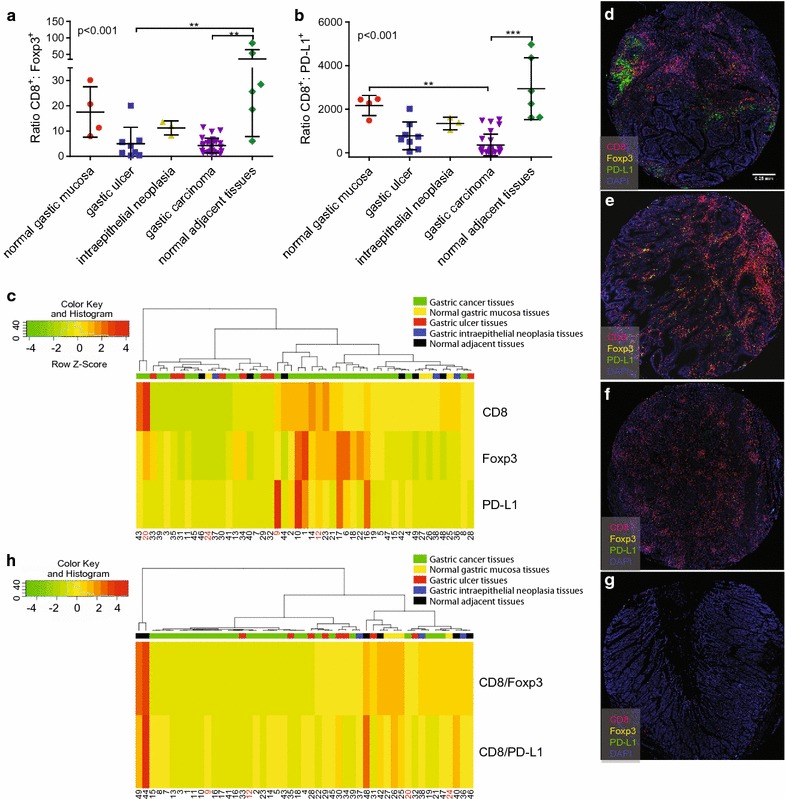
Quantification of the CD8^+^T:Foxp3 and CD8^+^T:PD-L1 ratios, heatmap generated in Rstudio, and 4-color mIHC images (× 10). **a** CD8a^+^:Foxp3^+^ ratio. **b** CD8^+^T:PD-L1^+^ ratio. **c** Hierarchical clustering of CD8^+^T, Foxp3 and PD-L1. **d** Scanned image of Sample 9 (10 × ). **e** Scanned image of Sample 12 (× 10). **f** Scanned image of Sample 24 (× 10). **g** Scanned image of Sample 20 (× 10). All images were acquired on a Nikon C1 confocal microscope. **h** Hierarchical clustering of CD8^+^T:Foxp3 and CD8^+^T:PD-L1. The top region represents the hierarchical clustering results. The number in the lower region represents the sample number

Based on the above results, CD8^+^T is insufficient for predicting the ability to generate TILs. However, when we evaluated the relative proportion of CD8^+^T and Foxp3^+^ cells, the ratio was highly significant (p < 0.05) among different gastric tissues, and the normal adjacent tissues possessed a significantly higher ratio of CD8^+^T and Foxp3^+^ cells compared with gastric cancer and gastric ulcer tissues (Fig. 5a). When we calculated the ratio of CD8^+^ and PD-L1^+^ cells, the ratios observed in normal adjacent tissues were also significantly higher than those in gastric cancer and normal gastric mucosa tissues (Fig. 5b), suggesting potential immunosuppressive roles for Foxp3 and PD-L1.

To dissect the immune phenotypes and cell interactions among CD8^+^T, Foxp3^+^ and PD-L1 in the microenvironment, heatmap and clustering analyses were performed to assess the potential correlation between different gastric diseases. First, we examined the 49 samples to see their clustering results based on the of CD8^+^T, Foxp3 and PD-L1 expression. As shown in Fig. 5c, all the samples clustered into 3 groups: one group contained two special samples of gastric cancer (higher CD8 expression), another contained most of the gastric cancer tissues (higher Foxp3 and PD-L1 expression), and the third group contained different types of gastric tissues. The group containing most of the gastric cancer tissues possessed higher PD-L1 and Foxp3 levels. However, PD-L1 was expressed partly in gastric cancer tissue based on the results of Fig. 5d (sample 9, gastric cancer tissue). Some gastric cancer also expressed lower PD-L1 (Fig. 5e, sample 12, gastric cancer tissue and Fig. 5f, sample 20, gastric cancer tissue).

Therefore, the ratio of CD8:Foxp3 and CD8:PD-L1 was used as a signature to determine the hierarchical clustering results of the 49 samples (Fig. 5h). All of the samples were divided into three main groups. One main group contained most of the gastric cancer tissues and gastric ulcer tissues based on their low CD8/Foxp3 and CD8/PD-L1 expression, as shown in Fig. 5d, e. Other normal tissues and gastric disease tissues, which exhibited relatively high expression of CD8/Foxp3 and CD8/PD-L1, were clustered into another group (Fig. 5g, sample 24, normal gastric mucosa tissue), and two normal adjacent tissues comprised the third group.

### CD8, Foxp3 and PD-L1 signature in gastric cancer tissue

**Fig. 6:**
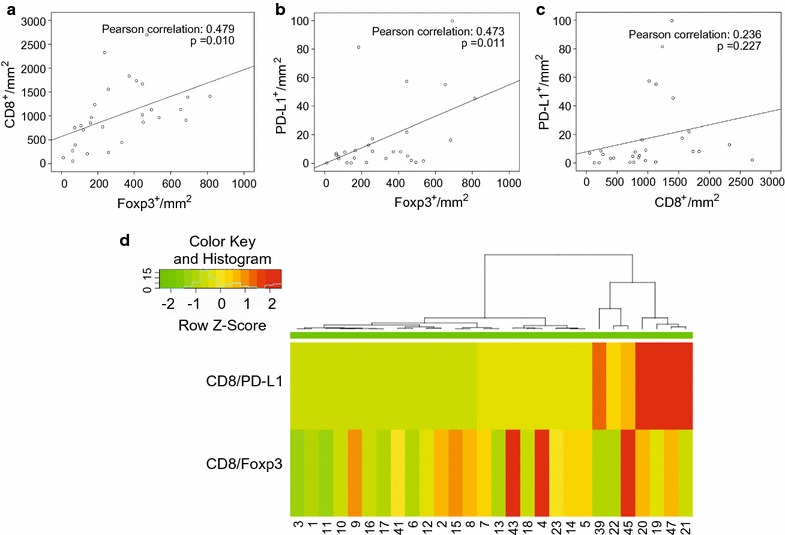
The relationship between the expression of CD8, Foxp3 and PD-L1 in gastric cancer tissues. **a** Pearson correlation of CD8 and Foxp3 (the correlation coefficient was 0.479, p < 0.01). **b** Pearson correlation of PD-L1 and Foxp3 (the correlation coefficient was 0.473, p < 0.05). **c** Pearson correlation of PD-L1 and CD8 (the correlation coefficient was 0.236, p > 0.05). **d** Hierarchical clustering of CD8^+^T:Foxp3 and CD8^+^T:PD-L1 in 28 gastric cancer samples. The number in the lower region represents the sample number

To dissect the immune phenotypes and cell interactions among CD8^+^T, Foxp3^+^ and PD-L1 in the microenvironment, we further aim to refine subgroups in gastric patients. The heatmap and clustering analyses were performed to assess the potential correlation between different gastric diseases. However, we cannot distinguish each disease immune phenotype by single marker CD8^+^T, Foxp3^+^ and PD-L1 clustering analyses (Fig. 5c). But when using the ratio of CD8:Foxp3 and CD8:PD-L1 for the hierarchical clustering, we were able to divide the samples into three main groups and correlated to the disease. One main group contained most of the gastric cancer tissues and gastric ulcer tissues based on their low CD8/Foxp3 and CD8/PD-L1 expression, as shown in Fig. 5h. Other normal tissues and gastric disease tissues, which exhibited relatively high expression of CD8/Foxp3 and CD8/PD-L1, were clustered into another group. The hierarchical clustering was also supported by additional Pearson correlation analysis in gastric cancer tissues. As shown in Fig. 6a–c, the correlation coefficient between the expression of CD8 and Foxp3 in gastric cancer tissues was 0.479 (p < 0.01). Moreover, the expression of Foxp3 was also high correlated with PD-L1 with the coefficient of 0.473 (p < 0.05). However, no significant relationship was found between the expression of CD8 and PD-L1 in gastric cancer tissues. Then we applied hierarchical clustering analysis in gastric cancer tissues (Fig. 6d). Based on the ratio of CD8:PD-L1, gastric cancer tissues were divided into three groups. However, after adding the ratio of CD8/Foxp3 for the further clustering analysis, more than 6–7 subtype gastric cancer immune phenotype formed, which indicated the complexity of classification. These more refined groups may help us to divide gastric cancer patients of the same clinical stage into different risk subgroups, which could potentially be useful for prediction and treatment guidelines of PD-L1 therapy.

## Discussion

We established a novel 4-color mIHC method that facilitates studying multiple parameters simultaneously in gastric disease tissues, which can illuminate important suppressive mechanisms within the tumor microenvironment [20]. This technology may help overcome the limitations of conventional single-color immunohistochemistry approaches used to classify patients based on the degree of their CD3 and CD8 T cell infiltration [21]. Here, our data revealed that the interactions between different immune populations serve as a better predictor compared with CD8 T cell density alone. Furthermore, by integrating this tool with unsupervised hierarchical analysis, we are able to observe the correlation patterns and signatures of CD8 and Foxp3 TIL densities and PD-L1 levels, which may indicate that PD-L1 regulates the immune response. This finding may be important in understanding the mechanisms of action of therapies and developing predictive biomarkers for more direct therapy.

Through the application of digital pathology tools for biomarker discovery and validation, CD8^+^ T cell infiltrates have been shown to have prognostic value in various types of cancer. High densities of CD8 and PD-L1 staining correlate with responses to anti-PD-1 immunotherapy agents in renal cell carcinoma (RCC), melanoma and non-small cell lung cancer (NSCLC) [22]. Patients with both high levels of T cell infiltrates and high PD-L1 expression in their tumors may fail to respond to anti-PD-L1 therapy. Complex tumor microenvironments are difficult to encapsulate with single markers such as CD8 and PD-L1. Thus, utilizing multiparametric analyses of immune checkpoints, including PD-L1, CD8 and Foxp3, to study the interactions between cell types may provide a more comprehensive view of immune phenotypes and signatures in the tumor microenvironment, which may help develop predictions and accurately stratify patients compared with CD8 alone.

The link between PD-L1 expression and immunotherapeutic outcomes can vary, possibly due to different IHC assays. Unresolved issues, including different staining protocols, different antibody protocols, and different scoring methods for identifying target cells as TCs, tumor-infiltrating immune cells (TIICs), etc., likely contribute to the unstable PD-L1 landscape. We used the E13LN clone from Cell Signaling Technologies, which demonstrates greater antibody sensitivity compared with other PD-L1 antibodies. We also optimized the experimental conditions to compare traditional IHC and 4-color mIHC using the same antibodies. Automatic identification of specific cells and tissue compartment types using trainable feature recognition algorithms was also implemented for our pattern and morphology analysis.

Immune responsiveness to the presence of a tumor is dependent on the proximity lymphocytes to the tumor. Endogenous CD8^+^ tumor-infiltrating T cells have been identified in a small series of patients with advanced gastric cancer [23]. These naturally occurring CD8^+^ TILs can specifically recognize autologous tumor-derived cells. However, in tumor regression during late-stage gastric cancers, TILs are rarely seen in the tumor microenvironment, suggesting that TILs are more common in early-stage disease which we selected stage II cancer patients and that advanced gastrointestinal malignancies are less immunogenic due to the selection pressure of some cancer cell during disease progression [24]. Multiple large studies have shown that increased PD-L1 expression correlates with worse prognosis, highlighting the prognostic power of PD-L1 expression in gastric cancers [25]. Elevated the expression of PD-L1 is associated with advanced stage, more nodal metastases and worse outcomes [26]. The PD-L1 pathway has become an important target in cancer immunotherapy. However, the prognostic value of PD-L1 for gastric cancer still remains controversial due to the complexity of the tumor and immune cells interaction. For this propose, to comprehensive evaluation of PD-L1 expression, Foxp3 and CD8^+^ T cells is necessary and superior to predicting these interactions.

Recent study showed that molecular classification dividing gastric cancer into four subtypes: (1) tumors positive for Epstein–Barr virus, which display recurrent *PIK3CA* mutations, (2) extreme DNA hypermethylation, (3) amplification of *JAK2*, *CD274* (*PD*-*L1*) and *PDCD1LG2* (*PD*-*L2*); (4) microsatellite unstable tumors (Microsatellite instability, MSI), which show elevated mutation rates, including mutations of genes encoding targetable oncogenic signalling proteins [27]. Those interesting data showed that PD-L1/2 expression was elevated in EBV-positive tumors by 9p amplifications, which were enriched in the EBV subgroup (15% of tumors). These results may indicate that those molecular classification associated with immune phenotype (checkpoint), will be worth to follow up in the association between checkpoint and expression of EBV, phenotype of MSI in a much larger number of gastric cancer tissues and also relative survival data in our future study.

The main aim of this study was to analyze the tumor microenvironment by analyzing 3 markers on 4-μm-thick gastric disease tissue sections. Overall, we found that the method was reproducible. Our data showed that PD-L1 was expressed in gastric ulcers, TCs and TIICs but not in normal gastric mucosa or other gastric intraepithelial neoplasia tissues. Furthermore, the ratios of CD8^+^T:Foxp3 and CD8^+^T:PD-L1 were suppressed in tumor tissues. Using the CD8^+^T:PD-L1 ratio, we were able to divide the samples into three class groups, and further integrating the CD8^+^T:Foxp3 ratio, which increased the complicity of immune phenotypes status, we defined 6–7 signatures and allowed the separation of gastric cancer patients at the same stage into different risk-group subsets (Fig. 6). Thus, increasing tumor PD-L1 expression correlated with response rate in a recent PD-1 inhibitor (KEYNOTE-012) phase I clinical trial, which supported a trend toward improved overall response rate (ORR) and progression-free survival (PFS) [28]. However, a few cases with lower responses also had high levels PD-L1 expression, suggesting that using the CD8^+^T:Foxp3 and CD8^+^T:PD-L1 ratios to define 6–7 clusters to separate gastric cancer patients into different risk subgroups may be useful for predicting and guiding PD-L1 therapy.

## Conclusions

In conclusion, mIHC allows us to better understand immune phenotypes in human gastric disease tissues. Our sub-analysis indicated that the ratios of cytotoxic T cells to regulatory T cells (CD8^+^T:Foxp3) and cytotoxic T cells to PD-L1 (CD8^+^T:PD-L1) tended to predict anti-tumor interactions in the microenvironment, indicating that the combination of CD8^+^T, Foxp3 and PD-L1 exhibits more predictive potential than PD-L1 expression or CD8^+^ TIL density alone. These finding will help identify situations in which PD-L1 blockade therapy may be useful for patients with gastric cancer.

## Additional file

**Additional file 1.** Supplemental methods, supplementary tables for patients. **Figure S1.** H&E staining of the two TMAs. **Figure S2.** Comparison of the results of ImageJ software and the score by pathologists.

## Abbreviations

mIHC: multiplexed immunohistochemistry
PD-L1: programmed death-ligand 1
CD8^+^T: cytotoxic T cells
TCs: tumor cells
TIICs: tumor-infiltrating immune cells
TIL: tumor-infiltrating lymphocyte
Tregs: regulatory T cells
HRP: horseradish peroxidase
DAB: 3, 3′-diaminobenzidine
RCC: renal cell carcinoma
NSCLC: melanoma and non-small cell lung cancer
ORR: overall response rate
PFS: progression-free survival
