# Supplementary material for: Understanding immune phenotypes in human gastric disease tissues by multiplexed immunohistochemistry
Source: J Transl Med. 2017 Oct 12;15:206. doi: 10.1186/s12967-017-1311-8 (PMC5639762; doi:10.1186/s12967-017-1311-8)
Supplement: Supplementary file 1 — Additional file 1. Supplemental methods, supplementary tables for patients. Figure S1. H&E staining of the two TMAs. Figure S2. Comparison of the results of ImageJ software and the score by pathologists. [file 12967_2017_1311_MOESM1_ESM.docx]

**SUPPLEMENTARY INFORMATION**

**1) Supplementary Methods**

**2) Supplementary Tables (Table S1)**

**3) Supplementary Figures (Figure S1)**

**Supplemental Methods**

**1. mIHC methods**

Firstly, optimize the antibody concentration for appropriate autoexposure times for single staining. After optimization, build and evaluate library using optimized single stain antibody, DAPI and unstained slides (Refer to Protocol for single staining of immunofluorescence). Select order of three antibodies incubation and perform multiplex stain with DAPI (Refer to Protocol for 4-color multiplex immunofluorescence). Then scan all the slides by Nikon C1 Confocal and images were analyzed by ImageJ software (Refer to Imaging and Software Analysis), the results were confirmed by two experienced pathologists (Supplementary Figure S2)

**2. Protocol for single staining of immunofluorescence**

**Step 1: Slide preparation**

1. Formalin-fixed-paraffin-embedded tumor samples

2. All the slides are put in oven at 60^o^C for 30 mins

**Step 2: Deparaffinization**

Xylene 3 times, each time 10 mins

100% Ethanol 2 times, each time 5 mins

75% Ethanol 3 mins

50% Ethanol 3 mins

25% Ethanol 3 mins

Wash all the slides in MQ-Water for 5 mins and TBST for another 5 mins

**Step 3: Antigen Retrieval**

Place slides in a plastic jar and fill it completely with sodium citrate buffer (pH 6.0). Loosely cover the jar with lid, place it in microwave oven at high heating level for about 50 s for boiling point, and then for additional 15 mins at low heating level. After that, cool down at room temperature for 30 mins. Circle the section with a PAP pen (Invitrogen, USA) and wash the slides with MQ-Water and TBST for 5 mins respectively.

**Step 4: Quench Endogenous Peroxidase**

Slides are incubated with 3% H_2_O_2_ (freshly made) for 10 mins at room temperature in a humid chamber. Then wash the slides with MQ-Water and TBST for 5 mins respectively.

**Step 5: Blocking**

Apply the blocking buffer (DAKO blocking buffer) for 10 min at room temperature in chamber.

**Step 6 Primary Antibody Incubation**

Tap off the blocking buffer and apply the primary antibody and also negative control (IgG) in chamber at room temperature for 1 h. Remove the antibody and wash with TBST 2 mins for 3 times.

**Step 7: Introduction of Secondary-HRP**

Apply HRP reagent (Cell signaling, USA) in chamber at room temperature for 30 min in dark. Use adequate reagent volume to cover the tissue section (about 100-300 µL per slide). Wash with TBST 3 x 2 mins.

**Step 8: Opal Signal Generation**

Drain off excess wash buffer and apply Opal working solution (PerkinElmer, USA), incubate at room temperature for 10 min. Wash with TBST 3 x 2 mins.

**Step 9: Mount**

Cover the slides with ProLong Gold Antifade Reagent with DAPI (Cell signaling, USA) and coverslip.

**3. Protocol for 4-color multiplex immunofluorescence**

**Step 1: Slide preparation**

1. Formalin-fixed-paraffin-embedded tumor samples

2. All the slides are put in oven at 60^o^C for 30 mins

**Step 2: Deparaffinization**

Xylene 3 times, each 10 mins

100% Ethanol 2 times, each 5 mins

75% Ethanol 3 mins

50% Ethanol 3 mins

25% Ethanol 3 mins

Wash all the slides in MQ-Water for 5 mins and TBST for another 5 mins

**Step 3: Antigen Retrieval**

Place slides in a plastic jar and fill it completely with sodium citrate buffer (pH 6.0). Loosely cover the jar with lid, place it in microwave oven at high heating level for about 50 s for boiling point, and then for additional 15 mins at low heating level. After that, cool down at room temperature for 30 mins. Circle the section with a PAP pen (Invitrogen, USA) and wash the slides with MQ-Water and TBST for 5 mins respectively.

**Step 4: Quench Endogenous Peroxidase**

Slides are incubated with 3% H_2_O_2_ (freshly made) for 10 mins at room temperature in a humid chamber. Then wash the slides with MQ-Water and TBST for 5 mins respectively.

**Step 5: Blocking**

Apply the blocking buffer (DAKO blocking buffer) for 10 min at room temperature in chamber.

**Step 6: Primary Antibody Incubation (CD8a)**

After that, tap off the blocking buffer and apply the primary antibody (CD8a, Cell Signaling, USA) and also negative control (IgG) in chamber at room temperature for 1 h. Remove the antibody and wash with TBST 2 mins for 3 times.

**Step 7: Introduction of Secondary-HRP**

Apply HRP mouse reagent (Cell signaling, USA) in chamber at room temperature for 30 min in dark. Use adequate reagent volume to cover the tissue section (about 100-300 µL per slide). Wash with TBST 3 x 2 mins.

**Step 8: Opal Signal Generation**

Drain off excess wash buffer and apply 670 Opal working solution (PerkinElmer, USA), incubate at room temperature for 10 min. Wash with TBST 3 x 2 mins.

**Step 9: Antigen Retrieval**

Place slides in a plastic jar and fill it completely with sodium citrate buffer (pH 6.0). Loosely cover the jar with lid, place it in microwave oven at high heating level for about 50 s for boiling point, and then for additional 15 mins at low heating level. After that, cool down at room temperature for 30 mins. Wash the slides with MQ-Water and TBST for 5 mins respectively.

**Step 10: Blocking**

Apply the blocking buffer (DAKO blocking buffer) for 10 min at room temperature in chamber.

**Step 11: Primary Antibody Incubation (Foxp3)**

After that, tap off the blocking buffer and apply the primary antibody (Foxp3, Cell Signaling, USA) and also negative control (IgG) in chamber at room temperature for 1 h. Remove the antibody and wash with TBST 2 mins for 3 times.

**Step 12: Introduction of Secondary-HRP**

Apply HRP rabbit reagent (Cell signaling, USA) in chamber at room temperature for 30 min in dark. Use adequate reagent volume to cover the tissue section (about 100-300 µL per slide). Wash with TBST 3 x 2 mins.

**Step 13: Opal Signal Generation**

Drain off excess wash buffer and apply 570 Opal working solution (PerkinElmer, USA), incubate at room temperature for 10 min. Wash with TBST 3 x 2 mins.

**Step 14: Antigen Retrieval**

Place slides in a plastic jar and fill it completely with sodium citrate buffer (pH 6.0). Loosely cover the jar with lid, place it in microwave oven at high heating level for about 50 s for boiling point, and then for additional 15 mins at low heating level. After that, cool down at room temperature for 30 mins. Wash the slides with MQ-Water and TBST for 5 mins respectively.

**Step 15: Primary Antibody Incubation (PD-L1)**

After that, tap off the blocking buffer and apply the primary antibody (PD-L1, Cell Signaling, USA) and also negative control (IgG) in chamber at room temperature for 1 h. Remove the antibody and wash with TBST 2 mins for 3 times.

**Step 16: Introduction of Secondary-HRP**

Apply HRP rabbit reagent (Cell signaling, USA) in chamber at room temperature for 30 min in dark. Use adequate reagent volume to cover the tissue section (about 100-300 µL per slide). Wash with TBST 3 x 2 mins.

**Step 17: Opal Signal Generation**

Drain off excess wash buffer and apply 520 Opal working solution (PerkinElmer, USA), incubate at room temperature for 10 min. Wash with TBST 3 x 2 mins.

**Step 18: Mount**

Cover the slides with ProLong Gold Antifade Reagent with DAPI (Cell signaling, USA) and coverslip.

**4. Imaging and Software Analysis**

1. Load a slide onto Nikon C1 confocal Microscope.

2. Create new protocol for the following use.

3. Adjust exposure for each channel by single staining slides and load up single color slides and take single image for each single-color control and unstained using the same exposure settings.

6. Build single color compensation library using the single-color control slides.

7. Use the single-color compensation library and scan the whole tissue of each sample.

8. Use image J to analysis the image.

9. For the analysis for CD8a and Fop3, the positive cell number per mm^2^ is used for further assessment while for PD-L1, average intensity is used.

10. After that two experienced pathologists scored all the slides, the average score by the two pathologists and the score got by ImageJ software are compared to confirm the rationality of ImageJ software analysis.

11. Merge and process data in Excel and perform relevant statistical analysis in Prism.

**Supplementary Tables**

Table S1 Detail information of 49 Samples on two TMA

| Sample name | TMA name | Map ID in TMA slide | Pathology Type | Gender | Age |
| --- | --- | --- | --- | --- | --- |
| Sample 1 | TMA1 | A1 | Gastric carcinoma | M | 81 |
| Sample 2 | TMA1 | A2 | Gastric carcinoma | M | 75 |
| Sample 3 | TMA1 | A3 | Gastric carcinoma | M | 57 |
| Sample 4 | TMA 1 | A6 | Gastric carcinoma | F | 64 |
| Sample 5 | TMA 1 | A7 | Gastric carcinoma | M | 76 |
| Sample 6 | TMA 1 | A8 | Gastric carcinoma | M | 59 |
| Sample 7 | TMA 1 | B3 | Gastric carcinoma | M | 75 |
| Sample 8 | TMA 1 | B4 | Gastric carcinoma | M | 69 |
| Sample 9 | TMA 1 | B5 | Gastric carcinoma | M | 63 |
| Sample 10 | TMA 1 | B6 | Gastric carcinoma | F | 77 |
| Sample 11 | TMA 1 | B7 | Gastric carcinoma | M | 56 |
| Sample 12 | TMA 1 | B8 | Gastric carcinoma | F | 75 |
| Sample 13 | TMA 1 | C1 | Gastric carcinoma | F | 80 |
| Sample 14 | TMA 1 | C3 | Gastric carcinoma | M | 58 |
| Sample 15 | TMA 1 | C5 | Gastric carcinoma | M | 52 |
| Sample 16 | TMA 1 | C6 | Gastric carcinoma | M | 59 |
| Sample 17 | TMA 1 | C8 | Gastric carcinoma | F | 60 |
| Sample 18 | TMA 1 | D1 | Gastric carcinoma | F | 61 |
| Sample 19 | TMA 1 | D2 | Gastric carcinoma | M | 51 |
| Sample 20 | TMA 1 | D3 | Gastric carcinoma | M | 70 |
| Sample 21 | TMA 1 | D4 | Gastric carcinoma | F | 77 |
| Sample 22 | TMA 1 | D5 | Gastric carcinoma | M | 68 |
| Sample 23 | TMA 1 | D6 | Gastric carcinoma | F | 66 |
| Sample 24 | TMA 2 | A1 | Normal gastric tissue | M | 49 |
| Sample 25 | TMA 2 | A2 | Normal gastric tissue | M | 65 |
| Sample 26 | TMA 2 | A3 | Normal gastric tissue | M | 64 |
| Sample 27 | TMA 2 | A6 | Normal gastric tissue | M | 70 |
| Sample 28 | TMA 2 | A7 | Gastric ulcer | M | 42 |
| Sample 29 | TMA 2 | A8 | Gastric ulcer | F | 20 |
| Sample 30 | TMA 2 | B1 | Gastric ulcer | M | 70 |
| Sample 31 | TMA 2 | B2 | Gastric ulcer | F | 40 |
| Sample 32 | TMA 2 | B3 | Gastric ulcer | M | 42 |
| Sample 33 | TMA 2 | B4 | Gastric ulcer | M | 55 |
| Sample 34 | TMA 2 | B5 | Gastric ulcer | M | 40 |
| Sample 35 | TMA 2 | B6 | Gastric ulcer | M | 43 |
| Sample 36 | TMA 2 | B8 | Gastric intraepithelial neoplasia | M | - |
| Sample 37 | TMA 2 | C1 | Gastric intraepithelial neoplasia | M | - |
| Sample 38 | TMA 2 | C2 | Gastric intraepithelial neoplasia | M | - |
| Sample 39 | TMA 2 | C3 | Gastric carcinoma | M | 55 |
| Sample 40 | TMA 2 | C4 | Normal adjacent tissue | M | 55 |
| Sample 41 | TMA 2 | C5 | Gastric carcinoma | M | 63 |
| Sample 42 | TMA 2 | C6 | Normal adjacent tissue | M | 63 |
| Sample 43 | TMA 2 | C7 | Gastric carcinoma | F | 77 |
| Sample 44 | TMA 2 | C8 | Normal adjacent tissue | F | 77 |
| Sample 45 | TMA 2 | D1 | Gastric carcinoma | M | 64 |
| Sample 46 | TMA 2 | D2 | Normal adjacent tissue | M | 64 |
| Sample 47 | TMA 2 | D3 | Gastric carcinoma | M | 59 |
| Sample 48 | TMA 2 | D4 | Normal adjacent tissue | M | 59 |
| Sample 49 | TMA 2 | D6 | Normal adjacent tissue | M | 67 |


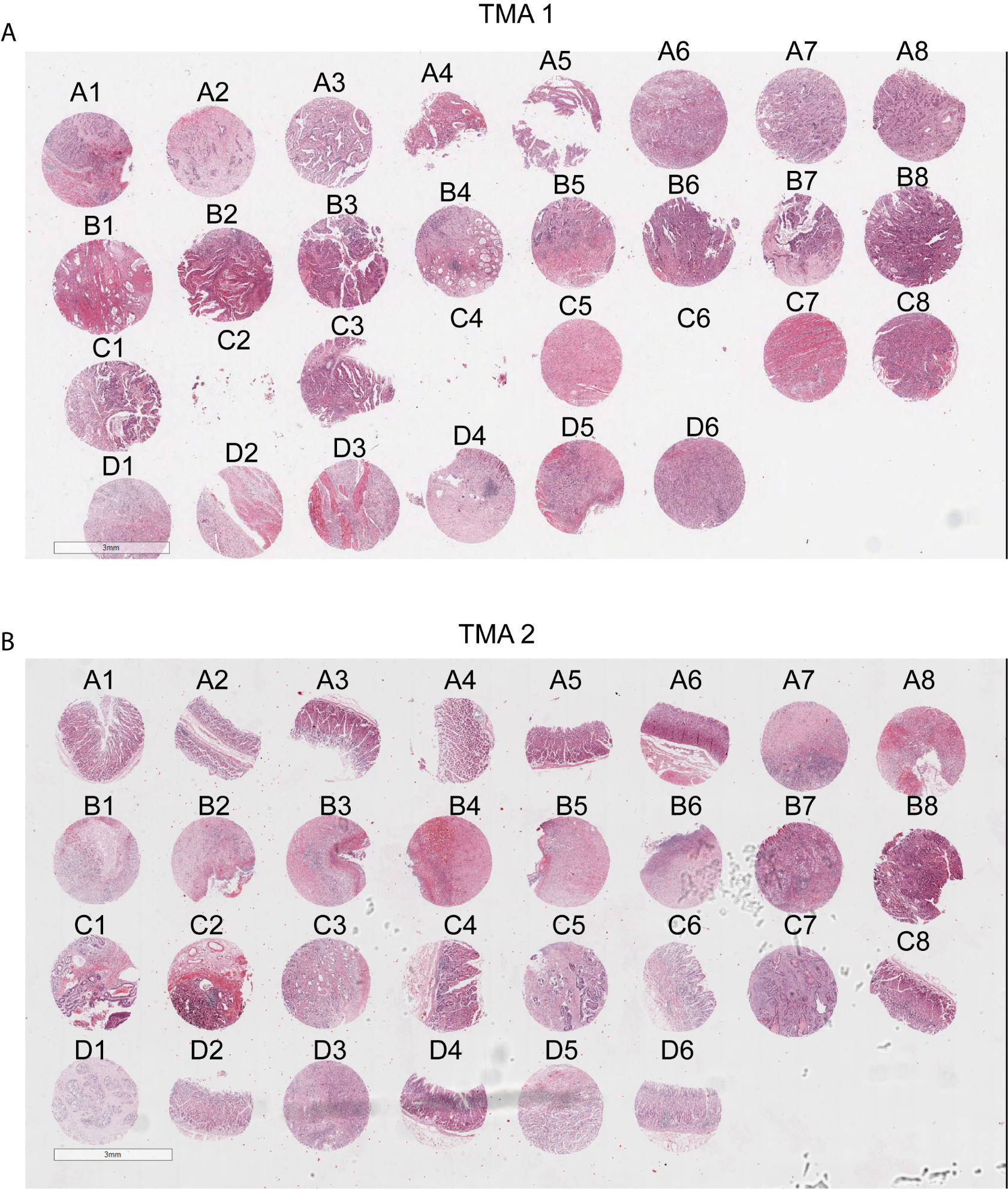


Figure S1 H&E staining of the two TMAs

A

B

C

Figure S2 Comparison of the results of ImageJ software and the score by pathologists
